# Inhibition of Oncogenic Transcription Factor REL by the Natural Product Derivative Calafianin Monomer 101 Induces Proliferation Arrest and Apoptosis in Human B-Lymphoma Cell Lines

**DOI:** 10.3390/molecules20057474

**Published:** 2015-04-23

**Authors:** Alan T. Yeo, Spandan Chennamadhavuni, Adrian Whitty, John A. Porco, Thomas D. Gilmore

**Affiliations:** 1 Department of Biology, Boston University, Boston, MA 02215, USA; E-Mail: alanyeo@bu.edu; 2 Department of Chemistry, Boston University, Boston, MA 02215, USA; E-Mails: schennam@gmail.com (S.C.); whitty@bu.edu (A.W.); porco@bu.edu (J.A.P.J.); 3 Center for Molecular Discovery (BU-CMD), Boston University, Boston, MA 02215, USA

**Keywords:** c-Rel, REL, NF-kappaB, chemical inhibitor, B-cell lymphoma, calafianin, natural product

## Abstract

Increased activity of transcription factor NF-κB has been implicated in many B-cell lymphomas. We investigated effects of synthetic compound calafianin monomer (CM101) on biochemical and biological properties of NF-κB. In human 293 cells, CM101 selectively inhibited DNA binding by overexpressed NF-κB subunits REL (human c-Rel) and p65 as compared to NF-κB p50, and inhibition of REL and p65 DNA binding by CM101 required a conserved cysteine residue. CM101 also inhibited DNA binding by REL in human B-lymphoma cell lines, and the sensitivity of several B-lymphoma cell lines to CM101-induced proliferation arrest and apoptosis correlated with levels of cellular and nuclear REL. CM101 treatment induced both phosphorylation and decreased expression of anti-apoptotic protein Bcl-X_L_, a REL target gene product, in sensitive B-lymphoma cell lines. Ectopic expression of Bcl-X_L_ protected SUDHL-2 B-lymphoma cells against CM101-induced apoptosis, and overexpression of a transforming mutant of REL decreased the sensitivity of BJAB B-lymphoma cells to CM101-induced apoptosis. Lipopolysaccharide-induced activation of NF-κB signaling upstream components occurred in RAW264.7 macrophages at CM101 concentrations that blocked NF-κB DNA binding. Direct inhibitors of REL may be useful for treating B-cell lymphomas in which REL is active, and may inhibit B-lymphoma cell growth at doses that do not affect some immune-related responses in normal cells.

## 1. Introduction

Many cancer cell types show high levels of constitutive activity of NF-κB transcription factors (p50, p52, RelA/p65, c-Rel/REL and RelB) [1]. For example, *REL* gene amplifications occur in diffuse large B-cell lymphoma (DLBCL), Hodgkin’s lymphoma and follicular lymphoma [2], and overexpression of wild-type and mutant forms of human REL can transform lymphoid cells in culture [3,4]. Moreover, inhibition of REL can arrest the growth of B-lymphoma cell lines *in vitro* [5,6,7].

All NF-κB transcription factors have a conserved N-terminal domain called the Rel Homology Domain (RHD), which is required for dimerization and DNA binding. The NF-κB superfamily can be divided into two subfamilies—Rel proteins (c-Rel, p65, RelB) and NF-κB proteins (p50, p52)—based on sequence similarity within the RHD, as well as in sequences C-terminal to the RHD [8]. The five NF-κB subunits can form homodimers and heterodimers, which can differentially affect target gene expression. Classical NF-κB activation is characterized by activation of p50, p65 and/or c-Rel complexes, whereas activation of the alternative NF-κB pathway consists primarily of induction of p52/RelB heterodimers [8,9].

Most normal cells have low basal levels of nuclear NF-κB DNA-binding activity. Activation of NF-κB generally proceeds through a cytoplasmic cascade in which activated IκB kinase (IKK) phosphorylates the direct NF-κB inhibitor IκB, which is then proteolytically degraded allowing NF-κB to enter the nucleus in an active DNA-binding form [8]. A multitude of extracellular factors, including many immune cell regulators such as cytokines, activate NF-κB, enabling it to turn on target gene transcription [9]. Many B-lymphoma cells have constitutively high levels of active, nuclear NF-κB DNA binding due to mutations in positive and negative regulators of NF-κB signaling or to autocrine signaling [10].

Many compounds that limit NF-κB activity have been described, and inhibitors of almost every step of the NF-κB pathway are known [11]. Because of its role in chronic inflammation and in cancer cell proliferation and survival, the NF-κB signaling pathway has often been proposed as a therapeutic target. Nevertheless, because of NF-κB’s role in normal cell function in a range of tissue and cell types, inhibitors that broadly ablate NF-κB signaling have not shown substantial therapeutic value [12].

Distinct biological functions for NF-κB subunits have been demonstrated in mouse developmental and knockout (KO) studies. p50 and p65 are necessary for development of secondary lymphoid organs and the liver, as judged by the phenotypes of *nfkb1* and *rela* KO mice, respectively [13,14]. c-Rel is primarily expressed at high levels in a subset of lymphoid cell types, and is required for immune-based activation and proliferation of B and T cells [2,13,14]. Therefore, c-Rel KO mice have low levels of induced immune cell activity, but these mice are otherwise healthy [13,14]. Moreover, c-Rel KO mice are refractory to certain induced models of inflammatory disease, such as collagen-induced arthritis [15]. Thus, c-Rel-specific inhibitors might be expected to be more favorable in a clinical setting than pan-NF-κB inhibitors or compounds targeting other NF-κB subunits.

In this report, we have characterized a compound (CM101) that preferentially inhibits DNA binding by REL and p65. Furthermore, we show CM101 inhibits the proliferation of human B-lymphoma cell lines with high levels of REL, and induces apoptosis in these cells through a mechanism that may involve inhibition of REL-dependent up-regulation of the anti-apoptotic gene/protein Bcl-X_L_. Nevertheless, induced activation of NF-κB signaling is reasonably robust in macrophages in the presence of CM101 at concentrations that affect B-lymphoma cell growth and survival.

## 2. Results and Discussion

### 2.1. Calafianin Monomer (CM101) Preferentially Inhibits REL and p65 DNA-Binding Activity

**Figure 1:**
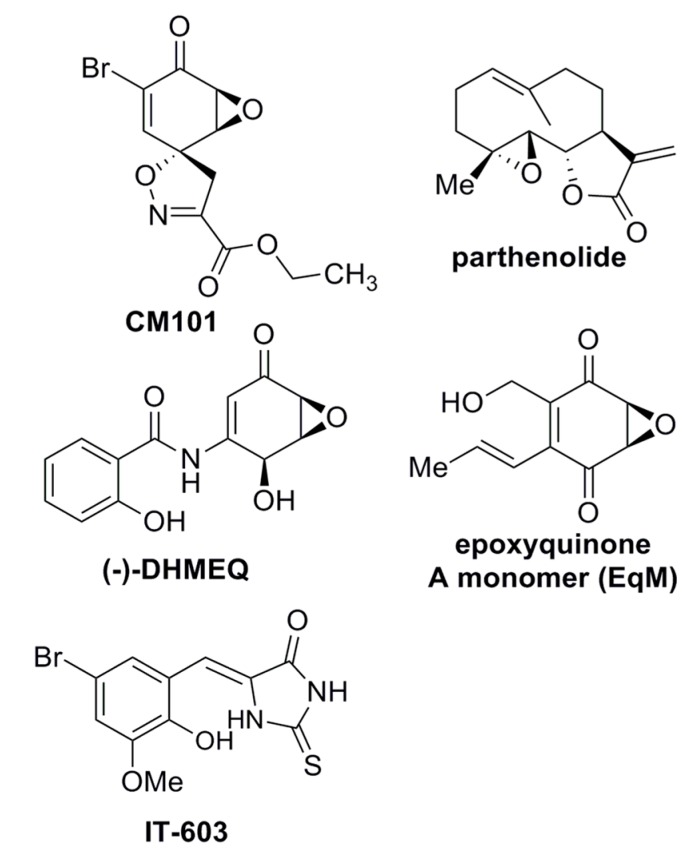
Structures of compounds with anti-REL activity. Shown are the chemical structures of CM101, parthenolide [18,19], DHMEQ [(−)-dehydroxymethylepoxquinomicin] [20], epoxyquinone A monomer [21], and IT-603 [22].

While screening for compounds that inhibit NF-κB signaling, we identified calafianin monomer (CM101) as a promising hit. CM101, the monomer unit of the spiroisoxazoline natural product [16] calafianin [17], shares chemical moieties (*i.e.*, epoxy ketone, α,β-unsaturated carbonyl) with other known NF-κB inhibitors such as parthenolide, DHMEQ, and epoxyquinone A monomer, but substantially differs in its overall chemical structure (Figure 1).

**Figure 2:**
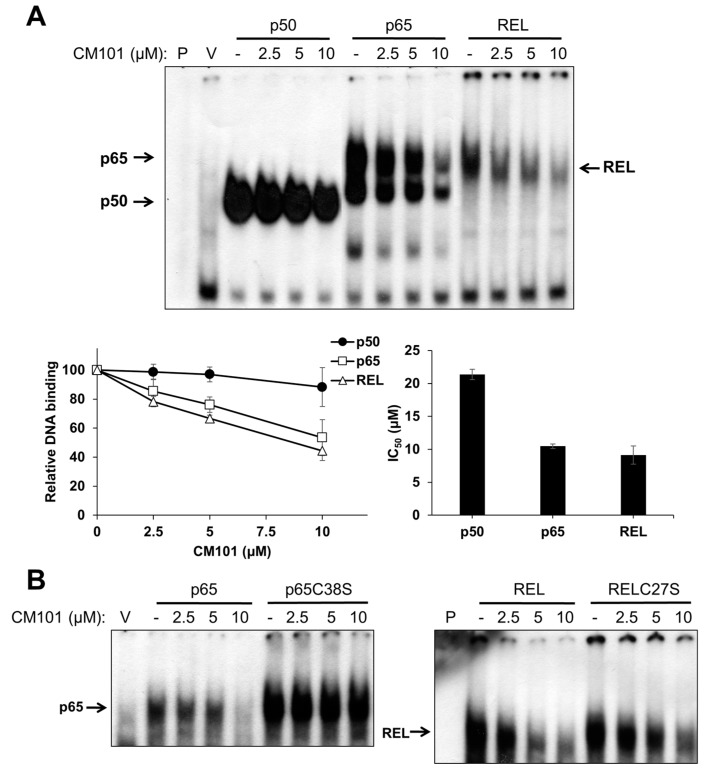
CM101 selectively inhibits REL and p65 but not p50 through covalent modification of cysteine residues. (**A**) 293 cells were transfected with expression vectors encoding FLAG-tagged human p50, p65 and REL, or with the empty vector (V). Transfected cells were treated with the indicated concentrations of calafianin monomer (CM101) for 2 h, extracts were made, and analyzed by EMSA with a κB-site probe. EMSA bands were detected by phosphorimaging and quantified. IC_50_ values were determined by plotting the band intensities at each concentration of CM101, using the control (no CM101) band intensity as 100%. All samples were treated with up to 20 µM CM101 (not shown), which was required to calculate the IC_50_ value for p50. Error bars indicate standard errors (three independent experiments); (**B**) 293 cells transfected with expression vectors encoding p65, p65C38S, REL or RELC27S were treated with CM101 and analyzed by EMSA as in (A). P, EMSA lane containing the probe alone.

To assess the effects of CM101 on the DNA-binding activity of canonical NF-κB subunits, human 293 cells were transfected with expression vectors for FLAG-tagged p50, p65, and REL. Two days later, transfected cells were treated with increasing concentrations of CM101 for 2 h, and then extracts were prepared and analyzed by electrophoretic mobility shift assay (EMSA) using a consensus NF-κB probe. The DNA-binding activities of p65 and REL were inhibited by CM101 at doses as low as 2.5 µM, whereas inhibition of p50 began at 10 µM (Figure 2A) The IC_50_ values for CM101 inhibition of p65 and REL DNA-binding activity were approximately 10 µM, whereas the IC_50_ for inhibition of p50 was greater than 20 µM.

Some NF-κB subunit inhibitors require a conserved Cys residue that is present in p65 (Cys38) and REL (Cys27) for maximal inhibition [6,18,19,20,21,23]. To determine whether inhibition by CM101 also requires this Cys residue, 293 cells transfected with expression vectors for wild-type or C38S mutant p65 and wild-type or C27S mutant REL were treated with increasing concentrations of CM101, and extracts were analyzed by EMSA. For both p65 and REL, the Cys mutants showed reduced inhibition of DNA binding by CM101 as compared to the respective wild-type proteins (Figure 2B). Western blotting of the same extracts under standard reducing SDS-PAGE conditions showed that treatment with CM101 converted a portion of wild-type p65 and REL to high molecular weight, covalently modified forms, whereas p50 cross-linking appeared to be restricted to a dimer (Figure S1A). Migration of the Cys-to-Ser mutant of REL was not affected by the same concentrations of CM101 (Figure S1B). Therefore, CM101-mediated inhibition of p65 and REL DNA binding may involve covalent modification at Cys38 and Cys27, respectively.

### 2.2. CM101 Inhibits Proliferation and Induces Apoptosis in B-Lymphoma Cell Lines

**Figure 3:**
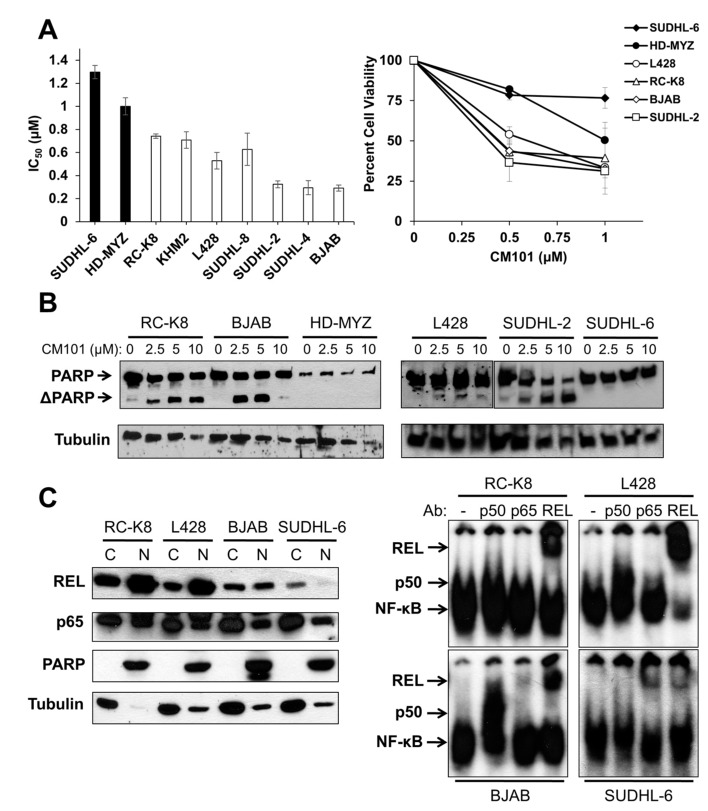
Sensitivity of human B-lymphoma cell lines to CM101 is correlated with active REL nuclear complexes. (**A**) A panel of B-lymphoma cell lines were seeded in triplicate at a cell number of 10^5^. Approximately 18 h later, cells were treated with increasing concentrations of CM101, incubated for an additional 72 h and cells were then counted. Cell numbers are relative to methanol-treated control cells (left panel). IC_50_ values were calculated by plotting the relative cell numbers at each concentration of CM101, which were normalized to cells incubated with no CM101 (100%) (see Figure S2A for the graph that was used to calculate IC_50_ values). Error bars indicate standard error. Right panel, MTT assays were performed and cell numbers were normalized to methanol-treated control cells; (**B**) The indicated B-lymphoma cell lines treated with various concentrations of CM101 for 24 h and cell extracts were analyzed for PARP cleavage by western blotting. The full-length and cleaved PARP (ΔPARP) bands are indicated. β-Tubulin western blotting was performed as a loading control; (**C**) Nuclear levels of REL were assessed from cytosolic and nuclear extracts prepared from RC-K8, L428, BJAB and SUDHL-6 cells. Extracts were analyzed by western blotting for REL and p65 (left panel). PARP and β-tubulin western blotting was performed to assess the purity of the nuclear and cytoplasmic fractions, respectively. EMSAs were performed on nuclear extracts with no antibody or antibodies for p50, p65 or REL for supershift analyses (right panel).

REL has been previously shown to be required for the proliferation of several B-lymphoma cell lines [2,14]. Because of CM101’s ability to inhibit REL DNA binding, we assessed the effect of CM101 on the proliferation and survival of human B-lymphoma cell lines. To do this, we treated nine human B-lymphoma cell lines with increasing concentrations of CM101 for 72 h and then counted cells at this time point. CM101 reduced cell proliferation in the different cell lines to varying degrees (Figure S2A). Based on the IC_50_ values for inhibition of cell proliferation by CM101 (Figure 3A), the nine B-lymphoma cell lines were separated into relatively resistant cell lines (defined as ones with IC_50_ ≥; 0.9 µM; SUDHL-6, HD-MYZ) and relatively sensitive cell lines (with the IC_50_ < 0.9 µM; RC-K8, KMH2, L428, SUDHL-8, SUDHL-2, SUDHL-4, BJAB). As a second measure of the effect of CM101 on lymphoma cells, we demonstrated that CM101 also had lesser effects on the cell viability of resistant SUDHL-6 and HD-MYZ cells as judged by an MTT assay (Figure 3A), which measures the activity of mitochondrial oxidoreductases that are associated with cell viability. In contrast, CM101 greatly reduced cell viability in all of the sensitive B-lymphoma cell lines.

We have previously shown that cleavage of the caspase-3 substrate poly(ADP-ribose) polymerase (PARP) is a reliable indicator of apoptosis in many of these same B-lymphoma cell lines [6,21,24]. To determine whether CM101 can induce apoptosis in B-lymphoma cell lines, four sensitive and two resistant B-lymphoma cell lines were treated with increasing concentrations of CM101 for 24 h, and cleavage of PARP was monitored by western blotting (Figure 3B). CM101 induced cleavage of PARP in the sensitive SUDHL-2, BJAB, and RC-K8 cell lines and to a lesser extent in L428 cells, but CM101 did not induce detectable PARP cleavage in resistant SUDHL-6 and HD-MYZ cell lines. In all six cell lines, CM101 induced apoptosis with potencies that had the same relative order as the IC_50_ values measured in the cell proliferation and MTT assays (Figure 3B). These results indicate that CM101 can selectively induce both cell growth arrest and apoptosis in certain B-lymphoma cell lines.

### 2.3. CM101 Inhibits REL DNA-Binding Activity in B-Lymphoma Cell Lines

To determine whether REL plays a role in the sensitivity of B-lymphoma cells to CM101, we first compared nuclear levels of REL in sensitive cell lines RC-K8, L428, and BJAB to levels in resistant SUDHL-6 cells. As a control for the integrity of the fractions, the blots were also probed for PARP (nuclear marker) and β-tubulin (cytosolic marker). Sensitive cell lines RC-K8 and L428 displayed high levels of nuclear REL, and BJAB had moderate levels of nuclear REL. Resistant SUDHL-6 cells had low levels of nuclear REL. In contrast, the levels of nuclear p65 did not differ among the four cell lines (Figure 3C). These results suggest that nuclear levels of REL, but not p65, correlate with the sensitivity of these cells to CM101.

To determine whether nuclear REL is active in these same four cell lines, an EMSA was performed on nuclear extracts. Constitutive κB site-binding activity was detected in all four cell lines, with the highest levels being in RC-K8 and L428 cells, lower levels in BJAB cells, and much lower levels in SUDHL-6 cells (Figure 3C). Using subunit-specific antibodies for supershifts, we found that RC-K8, L428 and BJAB EMSA complexes contained mostly REL, whereas SUDHL-6 complexes contained primarily p65 with lesser amounts of REL (Figure 3C). These results indicate that the sensitivity of B-lymphoma cells to CM101 correlates with the presence of nuclear REL DNA-binding activity, and not with the level or activity of p65.

**Figure 4:**
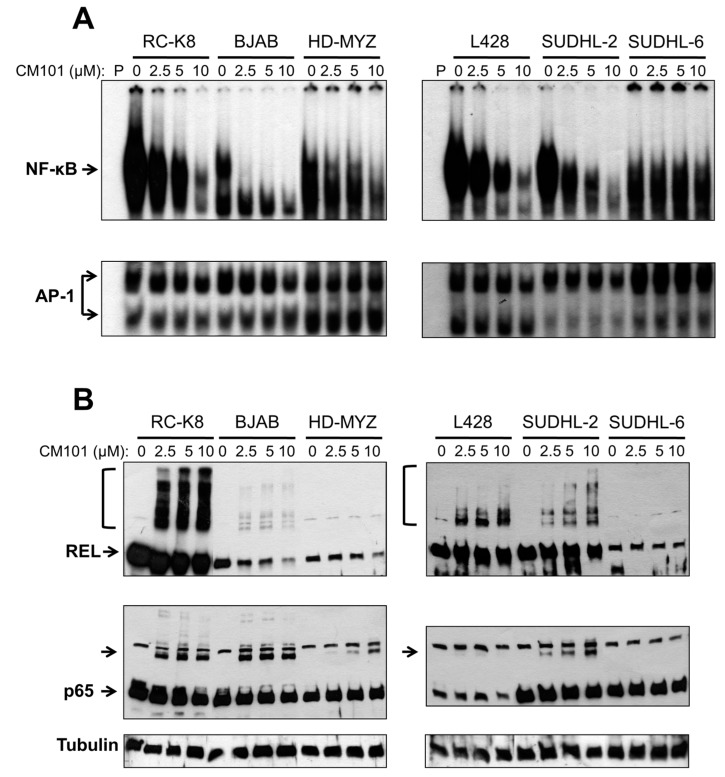
CM101 inhibits REL/NF-κB DNA binding in sensitive B-lymphoma cell lines. The named B-lymphoma cell lines were treated with the indicated concentrations of CM101 for 2 h, and extracts were made. (**A**) EMSAs were performed using a κB-site probe (top panels) or an AP-1 site probe (bottom panels). To make the amount of κB-site DNA-binding activity approximately equal for the various cell lines, the following amounts of cell extract were used: RC-K8, 5 µg; BJAB, 10 µg; HD-MYZ, 40 µg; L428, 10 µg; SUDHL-2, 15 µg; and SUDHL-6, 60 µg; (**B**) To assess the extent of cross-linking induced by CM101, extracts prepared from (A) were subjected to anti-REL or anti-p65 western blotting as indicated. REL and p65 indicate the monomer forms of the proteins. Brackets (for REL, top panels) or unlabeled arrow (p65 blots) designate higher molecular weight forms of the proteins. β-Tubulin western blotting was performed as a loading control (bottom panel).

We next investigated whether CM101 could inhibit constitutive NF-κB DNA binding in these lymphoma cells, and if so, whether CM101-induced inhibition of nuclear κB-site DNA binding correlated with the ability of CM101 to induce growth arrest and apoptosis. Sensitive (RC-K8 BJAB, L428 and SUDHL-2) and resistant (HD-MYZ, SUDHL-6) cell lines were treated with increasing concentrations of CM101 for 2 h, and extracts were analyzed by EMSA using a κB-site probe. The amount of cell extract used in the EMSAs was adjusted to make the amount of κB-site DNA-binding activity approximately equal for the various cell lines (see legend to Figure 4A). CM101 efficiently inhibited NF-κB DNA-binding activity in sensitive cell lines (BJAB, SUDHL-2, RC-K8, L428), but showed much less inhibition of NF-κB DNA-binding activity in resistant cells (HD-MYZ, SUDHL-6 cells), which had considerably less overall nuclear κB-site binding activity (Figure 4A). These results suggest that inhibition of NF-κB DNA binding by CM101 is responsible for its ability to induce cell growth arrest and apoptosis. Of note, CM101 did not inhibit transcription factor AP-1 DNA-binding activity at similar concentrations in these cell lines (Figure 4A). Thus, CM101 has a selective ability to inhibit NF-κB DNA binding among B-lymphoma cell lines, and B-lymphoma cell lines with high κB-site DNA-binding activity are sensitive to CM101-based inhibition.

To determine whether CM101 converts REL to higher molecular weight forms in B-lymphoma cells, as seen in 293 cells (Figure S1), western blotting was performed on the same lymphoma cell extracts used for the EMSAs in Figure 4A. REL was converted to high molecular weight forms in a dose-dependent manner in sensitive cell lines (RC-K8, L428, BJAB, SUDHL-2), but not in resistant cell lines (HD-MYZ, SUDHL-6) (Figure 4B). p65 had a lesser ability than REL to be converted to higher molecular weight forms. Additionally, the low basal κB-site DNA-binding activity in CM101-resistant SUDHL-6 and HD-MYZ cells may explain why they are not dependent on NF-κB signaling for survival.

### 2.4. Cellular Levels of REL Predict the Sensitivity of B-Lymphoma Cells to CM101, Which May Involve a REL-to-Bcl-X_L_ Pathway

**Figure 5:**
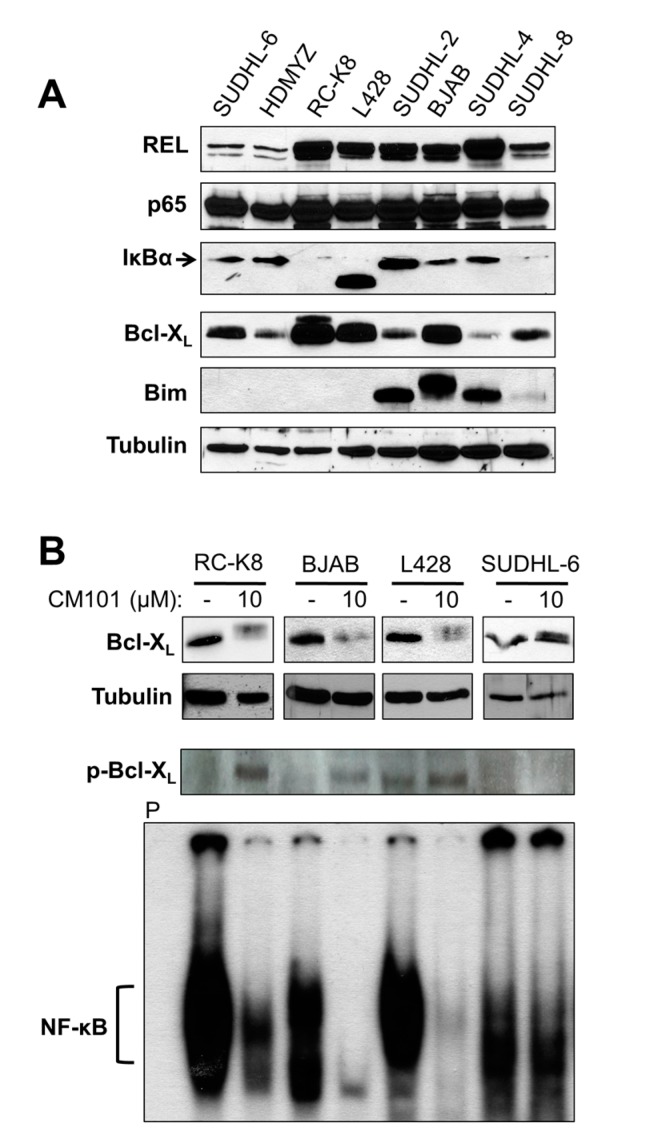
Total cellular levels of REL generally correlate with the sensitivity of B-lymphoma cells to CM101-induced apoptosis. (**A**) Extracts from the indicated B-lymphoma cell lines were probed by western blotting for REL, p65, IκBα, Bcl-X_L_, and Bim. β-Tubulin western blotting was performed as a loading control. (**B**) B-lymphoma cell lines RC-K8, L428, BJAB and SUDHL-6 were treated for 24 h with either the solvent methanol (−) or 10 μM CM101 (+). Anti-Bcl-X_L_, anti-phospho-Bcl-X_L_ and β-tubulin (loading control) western blotting was then performed on whole-cell extracts (upper three panels). In the lower panel, the indicated cell extracts (RC-K8, 10 µg; BJAB, 10 µg; L428, 15 µg, SUDHL-6, 80 µg) were analyzed by EMSA with a κB site-containing probe. P designates an EMSA lane containing the probe alone.

We have previously shown that the sensitivity of B-lymphoma cell lines to apoptosis-inducing compounds depends on levels of Bcl-2 family proteins [23,24]. Moreover, anti-apoptotic protein Bcl-X_L_ is the product of a REL target gene [25]. To identify proteins in the NF-κB and Bcl-2 families that could be important for the sensitivity of B-lymphoma cell lines to CM101, whole-cell extracts from the eight B-lymphoma cell lines were analyzed by western blotting for expression of NF-κB signaling proteins (REL, p65, IκBα) and of two major apoptotic regulatory proteins (anti-apoptotic Bcl-X_L_, pro-apoptotic Bim) (Figure 5A). Consistent with the EMSA results (Figure 4A), the total cellular levels of REL were low in CM101-resistant cell lines SUDHL-6 and HD-MYZ, whereas REL levels were high in all six CM101-sensitive cell lines. The p65 levels were similar in all eight cell lines. Moreover, all cell lines that were sensitive to CM101 had high levels of Bcl-X_L_ and/or high levels of Bim, whereas the two resistant cell lines, SUDHL-6 and HD-MYZ, had comparatively low levels of Bcl-X_L_ and no detectable Bim.

To determine if Bcl-X_L_ plays a role in CM101-induced apoptosis, we analyzed the effect of CM101 treatment on expression of Bcl-X_L_ in sensitive (RC-K8, L428, BJAB) and resistant (SUDHL-6) cells. Treatment with 10 µM CM101 for 24 h led to a reduction in Bcl-X_L_ levels in sensitive cell lines, whereas CM101 treatment did not affect the Bcl-X_L_ level in SUDHL-6 cells (Figure 5B). Moreover, CM101-sensitive cells showed the appearance of a slightly higher molecular weight form of Bcl-X_L_, which we hypothesized to be a phosphorylated form of Bcl-X_L_ seen in cells treated with other cytotoxic agents [26,27,28,29,30]. Indeed, anti-phospho-Bcl-X_L_ western blotting showed that treatment with CM101 led to increased levels of phosophorylated Bcl-X_L_ in sensitive (RC-K8, L428, BJAB) but not resistant (SUDHL-6) cells (Figure 5B). Furthermore, REL DNA-binding activity was inhibited by treatment with 10 µM CM101 for 24 h in sensitive cell lines, but κB-site binding was not inhibited in resistant SUDHL-6 cells (Figure 5B). These results show that down-regulation of both REL DNA-binding activity and Bcl-X_L_ expression are correlated with sensitivity to CM101-induced apoptosis in certain B-lymphoma cell lines.

### 2.5. Overexpression of Bcl-X_L_ or an Activated REL Mutant Can Protect B-Lymphoma Cells from CM101-Induced Apoptosis

**Figure 6:**
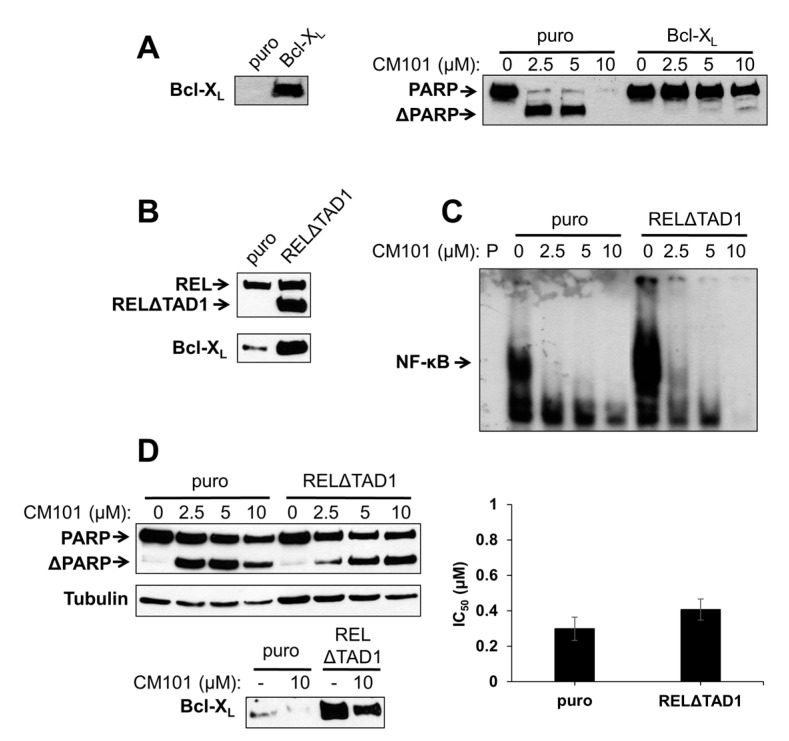
Ectopic expression of Bcl-X_L_ or an activated REL mutant can protect B-lymphoma cells from CM101-induced apoptosis. (**A**) SUDHL-2 cells were infected with a pMSCV-puro empty vector or the same vector encoding Bcl-X_L_. Stable pools were selected with puromycin. The left panel is a western blot confirming stable overexpression of retrovirally transduced Bcl-X_L_. In the right panel, SUDHL-2-puro and SUDHL-2-Bcl-X_L_ cells were treated with the indicated concentrations of CM101 for 24 h, extracts were prepared, and PARP cleavage was monitored by western blotting; (**B**) BJAB cells stably infected with pMSCV-puro empty vector or the same vector encoding RELΔTAD1 [4] were analyzed by western blotting for REL and Bcl-X_L_; (**C**) An EMSA with a κB-site probe using extracts of BJAB-puro and BJAB-RELΔTAD1 cells treated for 2 h with the indicated concentrations of CM101. P indicates probe alone; (**D**) BJAB-puro and BJAB-RELΔTAD1 cells were treated with the indicated concentrations of CM101 for 24 h, extracts were made, and PARP cleavage was monitored by western blotting. β-Tubulin western blotting was performed as a loading control. In the bottom left panel, Bcl-X_L_ levels were monitored by western blotting on extracts of BJAB-puro and BJAB-RELΔTAD1 cells treated for 24 h with either the solvent methanol (−) or 10 μM CM101 (+). In the right graph, BJAB-puro or BJAB-RELΔTAD1 cells were treated with increasing concentrations of CM101, and cells were counted 72 h later. IC_50_ values were calculated and plotted, as in Figure 3A.

To test directly whether Bcl-X_L_ can protect B-lymphoma cells from CM101-induced apoptosis, SUDHL-2 cells, which are sensitive to CM101-induced apoptosis and have low levels of Bcl-X_L_, were transduced with a retroviral vector containing the human Bcl-X_L_ gene or the parallel control vector. Pools of infected cells were selected with puromycin. Western blotting confirmed that Bcl-X_L_ was overexpressed in SUDHL-2 cells transduced with the Bcl-X_L_ vector as compared to control cells (Figure 6A). Control SUDHL-2-puro and experimental SUDHL-2-Bcl-X_L_ cells were then treated with increasing concentrations of CM101 for 24 h, and apoptosis was assessed by the extent of PARP cleavage. PARP cleavage was greatly reduced in SUDHL-2-Bcl-X_L_ cells as compared to control SUDHL-2-puro cells (Figure 6A). Thus, Bcl-X_L_ can protect SUDHL-2 B-lymphoma cells from CM101-induced apoptosis.

We have previously shown that overexpression of an activated REL mutant (RELΔTAD1) can enhance the transformed properties of BJAB cells and decrease their sensitivity to doxorubicin-induced apoptosis [4]. BJAB-RELΔTAD1 cells have increased levels of nuclear REL DNA-binding activity and consequently increased levels of Bcl-X_L_ [4,23] (Figure 6B). Therefore, we hypothesized that BJAB-RELΔTAD1 cells would be less sensitive to CM101-induced apoptosis than control BJAB-puro cells. Consistent with our previous findings [4], BJAB-RELΔTAD1 cells displayed higher levels of κB-site DNA binding than control BJAB-puro cells. CM101 inhibited κB-site DNA-binding activity in both BJAB-RELΔTAD1 and BJAB-puro cells (Figure 6C); however, because of the increased levels of κB site-binding activity in BJAB-RELΔTAD1 cells, the residual levels of DNA-binding activity after treatment with CM101 were higher in these cells than in control BJAB-puro cells (e.g., compare 2.5 µM and 5 µM lanes in Figure 6C).

To determine whether activated REL can protect cells from CM101-induced apoptosis, BJAB-puro and BJAB-RELΔTAD1 cells were treated with increasing concentrations of CM101, and PARP cleavage was monitored. Although PARP cleavage was observed in both cell types following treatment with CM101, the control cells were reproducibly more sensitive to CM101 (*i.e.*, PARP cleavage was seen at lower concentrations, e.g., 2.5 µM, of CM101 in BJAB-puro compared to BJAB-RELΔTAD1 cells) (Figure 6D). These results further suggest that enhanced REL activity can decrease sensitivity to CM101-induced apoptosis, possibly through up-regulation of Bcl-X_L_. Nevertheless, CM101 inhibited cell proliferation to a similar extent in both control BJAB-puro and BJAB-RELΔTAD1 (Figure 6D). Taken together, these results indicate that RELΔTAD1 protects BJAB cells from CM101-induced apoptosis, but not CM101-induced proliferation arrest.

To determine whether inhibition of REL could affect Bcl-X_L_ levels, control BJAB-puro and BJAB-RELΔTAD1 cells were treated with 10 µM CM101 for 24 h and cellular levels of Bcl-X_L_ were assessed. CM101 treatment resulted in decreased levels of Bcl-X_L_ in both BJAB-puro and BJAB-RELΔTAD1 cells (Figure 6D). However, the Bcl-X_L_ levels following CM101 treatment were higher in BJAB-RELΔTAD1 than BJAB-puro cells. These results suggest that the PARP cleavage observed in CM101-treated BJAB-RELΔTAD1 cells is due to down-regulation of Bcl-X_L_; however, apoptosis occurs to a lesser extent in BJAB-RELΔTAD1 cells compared to BJAB-puro cells due to the higher levels of Bcl-X_L_ in BJAB-RELΔTAD1 cells. These results indicate that up-regulation of Bcl-X_L_ can play a role in protecting B-lymphoma cells from CM101-induced apoptosis.

### 2.6. Upstream Components of NF-κB Signaling Can be Induced by Cytokines in the Presence of CM101

**Figure 7:**
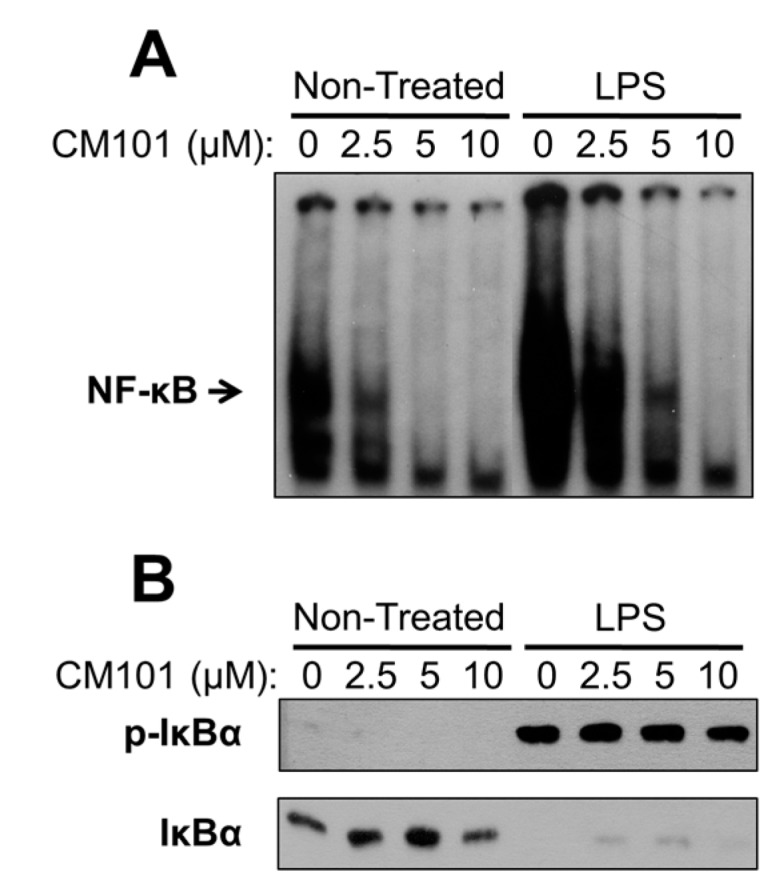
Activation of NF-κB signaling by LPS still occurs in the presence of CM101 in RAW264.7 macrophage cells. RAW264.7 macrophages were pretreated with the indicated concentrations of CM101 for 2 h and were then stimulated with 10 μg/mL LPS (Sigma-Aldrich, St. Louis, MO, USA) for 10 min. Extracts were made and subjected to (**A**) EMSA analysis with a κB-site probe or (**B**) western blotting for phospho-IκBα (p-IκBα) or total IκBα.

Because REL and p65 are preferential targets of CM101, we hypothesized that induced activation of NF-κB signaling would still occur in the presence of CM101 through utilization of upstream IKK activity. For these studies, we used RAW264.7 macrophages as a cell model for induced activation of NF-κB signaling. To assess the effect of CM101 on inflammation-like induction of NF-κB signaling, RAW264.7 cells were treated with increasing concentrations of CM101 for 2 h prior to stimulation with lipopolysaccharide (LPS). Extracts were then analyzed by EMSA using a κB-site probe (Figure 7A). In non-stimulated RAW264.7 cells, CM101 essentially completely inhibited NF-κB DNA binding at 5 µM. In LPS-treated cells, CM101 also dose-dependently inhibited NF-κB DNA-binding activity. Nevertheless, NF-κB DNA-binding activity was still readily detectable following LPS treatment in the presence of 5 µM CM101. In contrast to the effects of CM101 on NF-κB DNA binding, the phosphorylation and degradation of IκBα were minimally inhibited by CM101 in these cells even at 10 µM (Figure 7B). These results demonstrate that concentrations of CM101 that inhibit NF-κB subunit DNA binding can (1) still permit upstream activation of NF-κB signaling components (IKK→IκB degradation) and (2) allow partial activation of NF-κB DNA binding under conditions where basal NF-κB DNA binding is fully blocked.

### 2.7. Discussion

In this report, we have characterized a compound that directly and selectively inhibits the DNA-binding activity of cellular oncoprotein REL to cause B-lymphoma cell growth arrest and apoptosis. We show that the ability of CM101 to induce apoptosis in B-lymphoma cell lines correlated with the levels of constitutive REL DNA-binding activity and REL-controlled expression of anti-apoptotic Bcl-X_L_. In contrast, LPS-induced NF-κB pathway activation still occurs in the presence of CM101, supporting the conclusion that CM101 inhibits NF-κB pathway function by acting selectively at the level of the transcription factors. Therefore, CM101 belongs to a class of molecules that may be valuable for treatment of certain types of B-cell lymphoma.

CM101 has multiple electrophilic sites (indicated by bold arrows, Figure S3) that could enable it act as a covalent inhibitor [31]. Epoxy ketone pharmacophores have been shown to serve as irreversible covalent inhibitors by thiophilic ring opening with Cys residues on target proteins [32,33]. A probable mechanism includes initial attack of a cysteine thiol to the carbonyl carbon followed by epoxide ring opening via intramolecular 1,2 hydride shift to afford a dehydrated α-addition product (Figure S3). We have previously shown that other epoxyquinoids can cross-link REL to generate high molecular weight forms, and can inhibit REL DNA-binding activity [6]. CM101 acts on REL and p65, at least in part, through modification of a conserved Cys residue in a DNA-binding loop within the RHD. CM101 does not effectively inhibit p50 DNA-binding activity at concentrations where it inhibits REL and p65 DNA-binding activity, even though Cys is present at the analogous position in p50 and CM101 can crosslink p50 to a dimer. Flanking residues may affect the nature of the interaction of CM101-like compounds with p65 or REL vs p50, and the selective inhibitory activity of CM101 towards REL and p65 may depend on its ability to crosslink these NF-κB subunits to forms of higher molecular weight than the dimer. Of note, the specific inhibitory effect of DHMEQ on p65 can be reversed by changing five residues near to this Cys to those residues found in p50, which is not affected by DHMEQ [34]. Moreover, alterations in the DHMEQ structure can convert it from a p65 inhibitor to primarily an IKK inhibitor [35].

Cys-reactive inhibitors of NF-κB that act on multiple steps in the signaling pathway have been characterized by several groups [6,11,21]. CM101 is different because of its selectivity for inhibiting REL and p65 DNA-binding activity at concentrations at which upstream NF-κB signaling is unaffected. Namely, in many B-lymphoma cell lines CM101 substantially inhibits REL DNA-binding activity, arrests proliferation, and induces apoptosis at concentrations of 2.5–10 µM. However, in a macrophage cell line 10 µM CM101 had no detectable effect on upstream IKK activation as judged by LPS-induced phosphorylation and degradation of IκBα (Figure 7B). These results suggest that low concentrations of CM101 act primarily on REL and p65 rather than on p50 or the upstream IKK complex. Thus, CM101 and related compounds may have clinical relevance for inhibition of B-cell lymphomas that are dependent on constitutively high levels of nuclear REL DNA-binding activity. Moreover, REL and p65 have been shown to be required for optimal transformation by certain oncoproteins, including Ras [36,37], and *REL* was identified as a synthetic lethal gene in K-RAS mutant cancers [38]. Therefore, REL and p65 could also be drug targets in the many types of cancer that have activated Ras. Consistent with these observations, CM101 showed greater cell killing of Ras-transformed 3T3 cells than of control non-transformed 3T3 cells (Figure S2B).

NF-κB signaling has been commonly proposed as an anti-leukemia/lymphoma drug target. In particular, IKK inhibitors have been shown to be selectively toxic for the ABC subtype of diffuse large B-cell lymphoma (DLBCL), which has high levels of NF-κB activity and target gene expression [39,40,41]. However, in our study the sensitivity of lymphoma cell lines to inhibition by CM101 did not correlate with DLBCL subtype. That is, CM101 was effective at killing both ABC (RC-K8, SUDHL-2) and GCB (SUDHL-4, SUDHL-8, BJAB) cells, all of which have high levels of REL. Therefore, REL may be required for the growth/survival of certain lymphoma cell lines regardless of DLBCL subtype. In addition, nuclear REL staining has recently been shown to occur in both ABC and GCB tumors [42]. Thus, oncogenic NF-κB addiction cannot be solely identified through gene expression profiling, and may require NF-κB subunit profiling.

The RC-K8 and L428 cell lines both have inactivating mutations in the gene encoding IκBα [5,43,44]. Consequently, RC-K8 and L428 cells have abundant nuclear REL DNA-binding activity, and notably, undergo growth arrest upon ectopic expression of normal or super-repressor forms of IκBα, but not upon expression of dominant-negative IKK [5,39]. We show here that RC-K8 and L428 cells are both sensitive to inhibition of growth and REL DNA binding by CM101. Thus, direct inhibitors of REL, such as CM101, may be preferable to IKK inhibitors for those lymphomas that are refractory to IKK inhibitors due to mutations that activate the NF-κB pathway downstream of IKK.

Among the CM101-sensitive and -resistant B-lymphoma cell lines that we have characterized herein, only sensitive cell lines have high levels of REL and some of these cells also have high levels of Bcl-X_L_. The Bcl-X_L_ gene has been shown to be a direct target gene of REL [25]. Consistent with these observations, expression of the oncogenic RELΔTAD1 protein increased expression of Bcl-X_L_ in BJAB cells and made them slightly less sensitive to CM101-induced apoptosis. In addition, overexpression of Bcl-X_L_ dramatically protected SUDHL-2 cells from CM101-induced apoptosis. Taken together, these results confirm that Bcl-X_L_ can play an important role in the survival of B-lymphoma cells and that Bcl-X_L_ expression is dependent on transcription factor REL in some B-cell lymphomas. Thus, REL and/or Bcl-X_L_ should be suitable drug targets in some B-cell lymphomas.

SUDHL-6 and, to a lesser extent, HD-MYZ cells were both relatively resistant to CM101-induced growth arrest and apoptosis. Also, the total amount of constitutive NF-κB DNA-binding activity is low in both of these resistant cell lines (Figure 4A). In particular, SUDHL-6 cells have low amounts of active REL and its levels of Bcl-X_L_ were not affected by CM101. Therefore, Bcl-X_L_ is almost certainly not regulated by REL in SUDHL-6 cells, and SUDHL-6 does not appear to be an NF-κB/REL-addicted cell line. Moreover, phosphorylation of Bcl-X_L_, which is associated with sensitivity of several cell types to cytotoxic agents [26,27,28,29,30], did not occur in SUDHL-6 cells in response to CM101 treatment (Figure 5B). Although the SUDHL-6 cell line has been shown to be resistant to certain other toxic compounds, such as IKK and HDAC inhibitors [24,39], SUDHL-6 cells are not more resistant than other B-lymphoma cell lines to the cytotoxic effects of all compounds [45,46,47].

Inflammation is a key process for activation of the innate immune system [48]. Many kinase inhibitors used in targeted cancer therapies can compromise the responsiveness of the innate immune system [49], which may lessen their utility in clinical settings. NF-κB is a critical pathway for both inflammation and innate immune responses. Therefore, one concern for the use of NF-κB pathway inhibitors for cancer therapy is that such inhibitors would lessen immune system anti-tumor activity. CM101 appears promising in this regard because it selectively inhibits REL and p65 DNA-binding activity but not the activity of IKK. Thus, low concentrations of CM101-like compounds may still permit NF-κB DNA binding to be sufficiently induced in normal anti-tumor immune cells, while still inhibiting basal REL DNA-binding activity required for lymphoma cell growth. As such, compounds with CM101-like activity may be more effective anti-tumor agents because normal anti-tumor immune responses can still occur in the presence of lower concentrations of CM101-like compounds, in addition to the direct action of CM101 on tumor cell growth.

## 3. Experimental Section

### 3.1. Plasmids

Retroviral vector plasmids pMSCV-puro and pMSCV- Bcl-X_L_ (containing human Bcl-X_L_ cDNA subcloned into pMSCV-puro) have been described previously [23]. pcDNA-FLAG or pcDNA-based expression vectors for human p50, p65, p65C38S, REL and RELC27S have also been described previously [6,21].

### 3.2. Cell Culture, Transfections, and Chemical Treatment

Human 293, 293T, RC-K8, SUDHL-4, SUDHL-6, KMH2 and L428 cells and mouse 3T3 and RAW264.7 cells were grown in Dulbecco’s modified Eagle’s medium (DMEM) (Invitrogen, Carlsbad, CA, USA) supplemented with 10% heat-inactivated fetal bovine serum (FBS) (Biologos, Montgomery, IL, USA) (EF10). BJAB, BJAB-puro and BJAB-RELΔTAD1 cells [4] were grown in DMEM supplemented with 20% FBS (EF20). HD-MYZ, SUDHL-2, SUDHL-2-puro, SUDHL-2-Bcl-XL cells were grown in RPMI supplemented with 10% FBS (R10). SUDHL-8 were grown in RPMI supplemented with 20% FBS (R20). The human B-lymphoma cell lines used in this study have been characterized as follows: RC-K8, ABC-like DLBCL [5]; BJAB, SUDHL-6 and SUDHL-8, GCB-like DLBCL [39,50,51,52]; SUDHL-4, reported as both follicular lymphoma [53] and GCB-like DLBCL [39]; and L428, KMH2, and HDMYZ, Hodgkin’s lymphoma [43].

BJAB-puro and BJAB-RELΔTAD1 cell lines have been described previously [4]. For the creation of SUDHL-2 cell lines, virus stocks were generated by transfecting 293T cells with pMSCV-puro or pMSCV-Bcl-X_L_, plus helper plasmid pcL10a1, as described previously [23]. Approximately 48 h later, virus was harvested. Two mL of virus (in the presence of 8 μg/mL polybrene) was used to infect 10^6^ SUDHL-2 cells using the spin infection method [54]. Two days later, 1 μg/mL puromycin (Sigma, St. Louis, MO, USA) was added to the cells, and pools of infected cells were selected with puromycin for 2–4 weeks. CM101 is part of the BU-CMD chemical collection. The synthesis of CM101 was initiated by multi-gram synthesis of quinone monoketal **2** from the readily available compound 4-methoxyphenol (**1**) as previously described [17]. Enantioselective tartrate-mediated nucleophilic epoxidation provided epoxy ketone **3** that was subjected to Wittig olefination to afford the exocyclic vinyl epoxide **4**. 1,3-Dipolar cycloaddition of **4** with and *in situ*-prepared nitrile oxide in the presence of Zr(O*^t^*Bu)_4_ followed by acetal deprotection provided a mixture of *syn* and *anti* diastereomers (dr = 1:1) of CM101 in good to moderate yields (see Supporting Information, Scheme S1).

Twenty-four h prior to treatment with CM101, cells were plated into 35-mm 6-well plates at approximately 60%–70% confluency. Cells were then incubated with the indicated concentrations of compound or the solvent methanol for the indicated times.

For transfections, 293 cells were seeded such that they were approximately 60% confluent on the following day when transfections were performed using polyethylenimine (PEI; Polysciences, Warrington, PA, USA) [23]. Transfections were performed by incubating 5 μg of plasmid DNA in 30 μL PEI (1 mg/mL) and 400 μL of DMEM at room temperature. The DNA/PEI mixture was then added to 5 mL of EF10 and incubated with a 60-mm plate of 60%–70% confluent cells for 24 h. Transfected cells were then passed into 35-mm 6-well tissue culture plates, and cells were allowed to grow for 24 h prior to treatment.

### 3.3. Cell Proliferation and MTT Assays

Cell proliferation assays were performed as described previously [23]. Briefly, approximately 10^5^ cells were plated in 16-mm wells in 0.5 mL of media. After an 18-h recovery period, cells were treated with the indicated concentrations of CM101. For each treatment group, cells in triplicate wells were counted three days later. Cell numbers were normalized to the number of cells in the control, methanol-treated plates, and IC_50_ values were determined from those average cell counts. For MTT assays, cells were treated with MTT reagent (Sigma) and solubilized with SDS-acid solvent. Absorbance was read at 595 nm. Cell densities in treatment groups were normalized to cell densities in methanol-treated controls.

### 3.4. Biochemical Fractionation and Electrophoretic Mobility Shift Assays

Whole-cell and nuclear extracts were prepared and analyzed by EMSA as described previously [4,23]. Whole-cell extracts were prepared in AT lysis buffer [20 mM HEPES, pH 7.9, 150 mM NaCl, 1% (*v*/*v*) Triton X-100, 20% (*w*/*v*) glycerol, 1 mM EDTA, 1 mM EGTA, 20 mM NaF, 1 mM Na_4_P_2_O_7_, 1 mM DTT, 1 mM Na_3_VO_4_. 1 μg/mL PMSF, 1 μg/mL leupeptin, and 1 μg/mL pepstatin] [4,23]. Cytoplasmic and nuclear extracts were prepared as described previously [23,55]. Briefly, cells were washed with cold PBS, then resuspended in Buffer E (10 mM HEPES pH 7.9; 10 mM NaCl; 0.1 mM EDTA; 0.1 mM EGTA; 1 mM DTT; 1 mM Na_3_VO_4_, 1 μg/mL PMSF, 1 μg/mL leupeptin, and 1 μg/mL pepstatin), which was then supplemented with 10% IPEGAL to ~1%. Samples were centrifuged for 5 min at 800× *g* at 4 °C. The supernatant was removed as the cytoplasmic fraction. Nuclear pellets were resuspended in Buffer F (10 mM HEPES pH 7.9, 0.5 M NaCl, 1 mM EDTA, 1 mM EGTA, 1 mM DTT, 1 mM Na_3_VO_4_, 1 μg/mL PMSF, 1 μg/mL leupeptin, 1 μg/mL pepstatin), and samples were pelleted at top-speed in a microcentrifuge; the supernatant was used as nuclear extract. Nuclear or whole-cell extracts (5–80 μg, as described in Figure legends) were incubated with a 26-base pair radiolabeled κB-site probe (κB site: 5'-GGGAAATTCC-3'; ~200,000 cpm), 2 μg of poly (dI-dC) in binding buffer (25 mM Tris-HCl, pH 7.4, 100 mM KCl, 6.25 mM MgCl_2_, 0.5 mM EDTA, 0.5 mM DTT, 10% (*w*/*v*) glycerol) in a 50-μL reaction volume for 30 min at 30 °C. Supershifts were performed by an additional 1-h incubation with 1–2 μL of antibody on ice. Antisera for supershifts were as follows: anti-p50 (sc114x; Santa Cruz Biotechnology, Santa Cruz, CA, USA) and anti-p65 (#1226; gift of Nancy Rice); REL (#1507; Nancy Rice). Samples were electrophoresed on non-denaturing 5% polyacrylamide gels, and protein-DNA complexes were detected by autoradiography.

### 3.5. Western Blotting

Western blotting was performed essentially as described [23], using whole-cell, cytoplasmic or nuclear extracts that were prepared as described above. Samples containing equal amounts of total protein were separated on 7.5 or 10% SDS-polyacrylamide gels, and proteins were transferred to nitrocellulose membranes. Primary antisera (dilution; source) against the following proteins were used: PARP (1:500; Santa Cruz Biotechnology); Bcl-X_L_ (1:1000; Cell Signaling Technology, Danvers, MA, USA); Bim (1:1000; Cell Signaling Technology); REL C terminus (1:500; Nancy Rice); p65 C terminus (1:4000; Nancy Rice); IκBα (1:1000; Cell Signaling Technology); phospho-IκBα (1:1000; Cell Signaling Technology); phospho- Bcl-X_L_ (1:300; Millipore, Billerica, MA, USA); and β-tubulin (1:500; Santa Cruz Biotechnology). The appropriate horseradish peroxidase-labeled secondary antiserum was added, and complexes were detected by Supersignal Dura West chemiluminescence (Pierce Chemical, Rockford, IL, USA).

## 4. Conclusions

Our data suggest that CM101-like compounds that covalently modify REL to inhibit its DNA-binding activity represent a promising approach for the treatment of cancers (especially B-cell lymphomas) whose survival depends on active nuclear REL. Moreover, the viability and developmental integrity of c-*rel* KO mice [13,14] suggest that anti-REL therapeutics would not adversely affect non-immune cells in patients. Because of the deleterious effects of c-*rel* KO on B-cell proliferation [13], CM101 would likely affect the viability of normal B cells in addition to its effects on B-lymphoma cells. Indeed, many drugs that have therapeutic efficacy for treating B-cell lymphoma cause patients to become immune-compromised, which is an unfortunate but manageable condition (e.g., rituximab [56]). Of note, Shone *et al.* [22] reported compound IT-603 as a direct inhibitor of REL and showed that inhibition of REL can reduce graft-versus-host disease without affecting T cell-mediated anti-tumor activity in mouse models. Thus, much data indicate that REL may be a valuable target for certain types of anti-cancer and immunomodulatory therapies. Finally, our studies contribute new knowledge about covalent inhibitors of transcription factors as drug targets [31].

## Supplementary Materials

The Supplemental Information contains the following: Figure S1. CM101 selectively crosslinks p65 and REL to high molecular weight forms through cysteine residues; and Figure S2. CM101 inhibition of cell proliferation in a panel of B-lymphoma cell lines and in K-Ras-transformed mouse 3T3 cells. Scheme S1. Spiroisoxazoline (CM101) synthesis. Figure S3. Proposed mechanism of covalent inhibition and cysteine modification by CM101.

Supplementary materials can be accessed at: http://www.mdpi.com/1420-3049/20/05/7474/s1.
