# Supplementary material for: Inhibition of Oncogenic Transcription Factor REL by the Natural Product Derivative Calafianin Monomer 101 Induces Proliferation Arrest and Apoptosis in Human B-Lymphoma Cell Lines
Source: Molecules. 2015 Apr 23;20(5):7474–94. doi: 10.3390/molecules20057474 (PMC4863944; doi:10.3390/molecules20057474)
Supplement: Supplementary file 1 [file molecules-20-07474-s001.pdf]

Supplemental Information

The supplemental information contains three Supplemental Figures and Figure Legends, and one chemical synthesis scheme.

- **Figure S1.** CM101 selectively crosslinks p65 and REL to high molecular weight forms through cysteine residues.
- **Figure S2.** CM101 inhibition of cell proliferation in a panel of B-lymphoma cell lines and in K-Ras-transformed mouse 3T3 cells.
- **Scheme S1.** Spiroisoxazoline (CM101) synthesis.
- **Figure S3.** Proposed mechanism of covalent inhibition and cysteine modification by CM101.

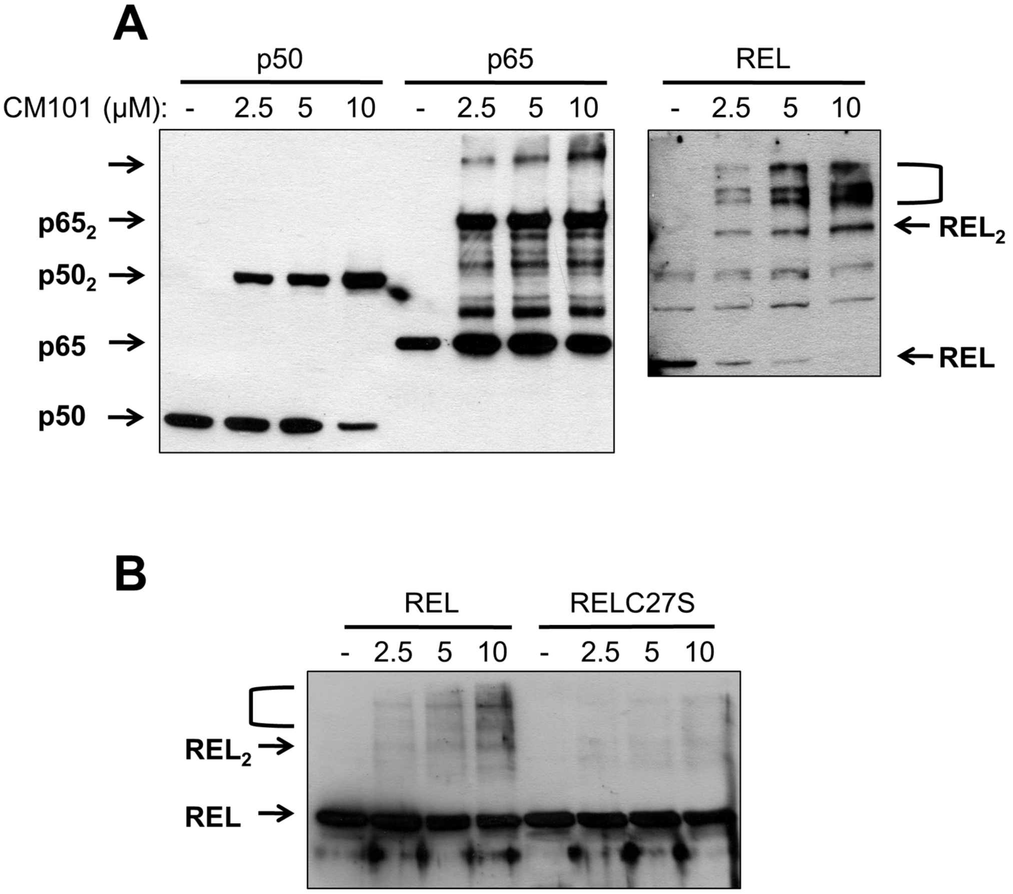

Figure S1. CM101 selectively crosslinks p65 and REL to high molecular weight forms through cysteine residues. (**A**) A293 cells were transfected with expression vectors encoding FLAG-tagged human p50, p65 and REL. Transfected cells were treated with the indicated concentrations of CM101 for 2 h, extracts were made, and anti-FLAG Western blotting was performed. The monomer forms of the proteins are indicated by their names. The likely dimers are indicated by $p50_2$, $p65_2$ and REL_2. The unlabeled left arrow (for p65) and right bracket (for REL) designate the higher molecular weight forms of these proteins. (**B**) A293 cells transfected with expression vectors encoding REL or RELC27S were treated with CM101 and analyzed by Western blotting for FLAG as in (A).

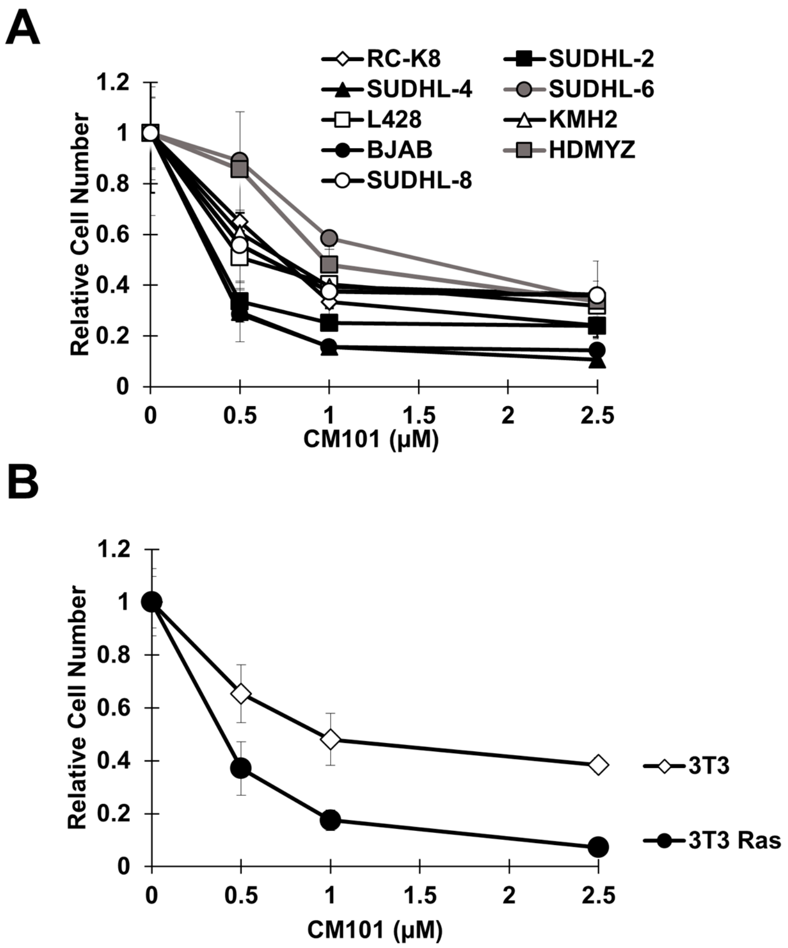

Figure S2. CM101 inhibition of cell proliferation in a panel of B-lymphoma cell lines and in K-Ras-transformed mouse 3T3 cells. (**A**) The indicated B-lymphoma cell lines were seeded in triplicate at a cell number of 10^5. Approximately 18 h later, cells were treated with increasing concentrations of CM101 and cells were counted 72 h later. Cell numbers are relative to methanol-treated control cells and plotted. (**B**) Wild-type 3T3 and Ras-transformed 3T3 cells were seeded, treated with CM101, and counted 72 h later as in (A).

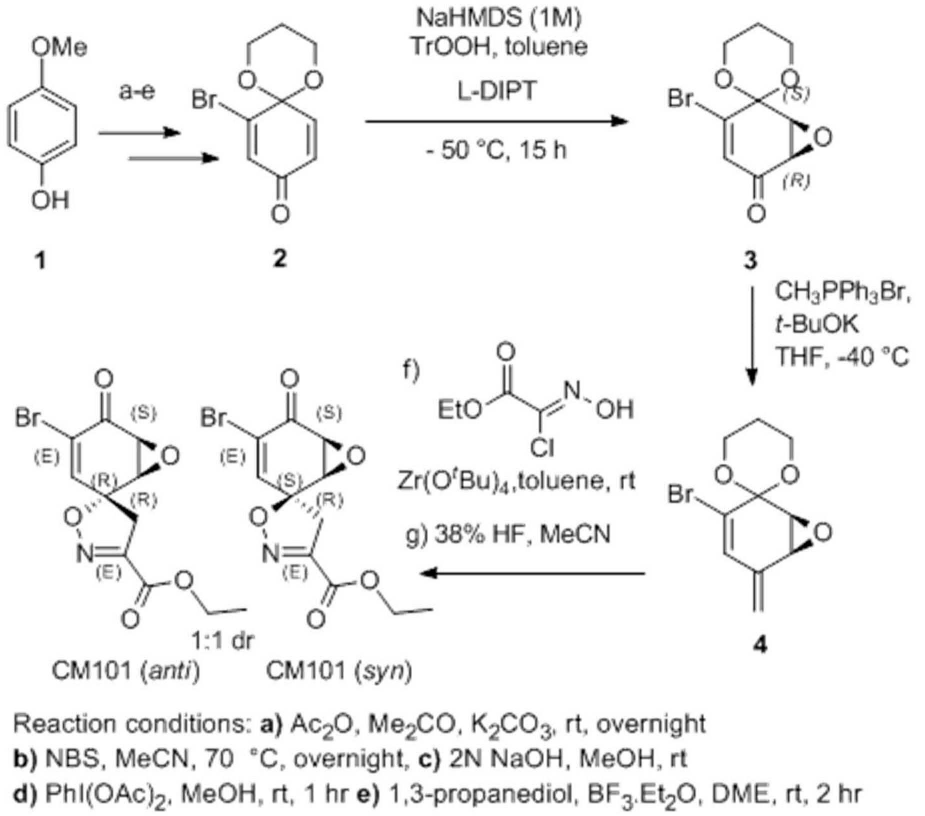

Scheme S1. Spiroisoxazoline (CM101) synthesis (as previously reported in [17]).

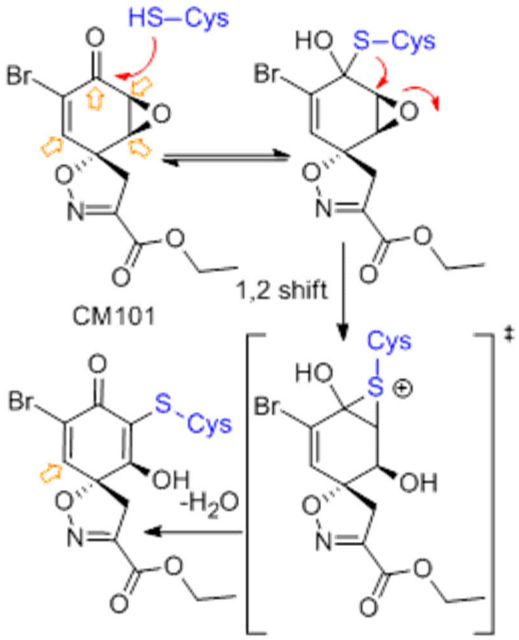

Figure S3. Proposed mechanism of covalent inhibition and cysteine modification by CM101. Modification of cysteine by CM101 in NF-κB target proteins likely includes initial attack of thiol to the carbonyl carbon followed by epoxide ring opening *via* intramolecular 1,2 hydride shift to provide a dehydrated α-addition product.
